# Diagnosis of osteoporosis from dental panoramic radiographs using the support vector machine method in a computer-aided system

**DOI:** 10.1186/1471-2342-12-1

**Published:** 2012-01-16

**Authors:** M S Kavitha, Akira Asano, Akira Taguchi, Takio Kurita, Mitsuhiro Sanada

**Affiliations:** 1 Graduate School of Engineering, Hiroshima University, 1-4-1 Kagamiyama, Higashi-Hiroshima, Hiroshima 739-8527, Japan; 2 Faculty of Informatics, Kansai University, 2-1-1 Ryozenji-cho, Takatsuki, Osaka 506-1095, Japan; 3 Department of Oral and Maxillofacial Radiology, Matsumoto Dental University, 1780 Hirooka-Gohara, Shiojiri, Nagano 399-0781, Japan; 4 Sanada Hospital, Minamimachi 3-13-21, Minami-ku, Hiroshima 734-0007, Japan

## Abstract

**Background:**

Early diagnosis of osteoporosis can potentially decrease the risk of fractures and improve the quality of life. Detection of thin inferior cortices of the mandible on dental panoramic radiographs could be useful for identifying postmenopausal women with low bone mineral density (BMD) or osteoporosis. The aim of our study was to assess the diagnostic efficacy of using kernel-based support vector machine (SVM) learning regarding the cortical width of the mandible on dental panoramic radiographs to identify postmenopausal women with low BMD.

**Methods:**

We employed our newly adopted SVM method for continuous measurement of the cortical width of the mandible on dental panoramic radiographs to identify women with low BMD or osteoporosis. The original X-ray image was enhanced, cortical boundaries were determined, distances among the upper and lower boundaries were evaluated and discrimination was performed by a radial basis function. We evaluated the diagnostic efficacy of this newly developed method for identifying women with low BMD (BMD T-score of -1.0 or less) at the lumbar spine and femoral neck in 100 postmenopausal women (≥50 years old) with no previous diagnosis of osteoporosis. Sixty women were used for system training, and 40 were used in testing.

**Results:**

The sensitivity and specificity using RBF kernel-SVM method for identifying women with low BMD were 90.9% [95% confidence interval (CI), 85.3-96.5] and 83.8% (95% CI, 76.6-91.0), respectively at the lumbar spine and 90.0% (95% CI, 84.1-95.9) and 69.1% (95% CI, 60.1-78.6), respectively at the femoral neck. The sensitivity and specificity for identifying women with low BMD at either the lumbar spine or femoral neck were 90.6% (95% CI, 92.0-100) and 80.9% (95% CI, 71.0-86.9), respectively.

**Conclusion:**

Our results suggest that the newly developed system with the SVM method would be useful for identifying postmenopausal women with low skeletal BMD.

## Background

Osteoporosis is a disease that develops asymptomatically in its early stages and is characterized by low bone mass and micro-architectural deterioration of bone tissue, thereby increasing the risk of fractures [1]. The incidence is higher in developed countries, primarily because they have a large elderly population. It is a major health problem in the Japanese elderly population as well and is estimated to affect approximately 12 million people [2]. A huge number of dental panoramic radiographs, offering greater opportunities for studying bones, are taken every year [3]. The features of osteoporosis can often be observed in the films of affected individuals, and there are significant relationships between the mandibular cortical bone quality, quantity and bone mineral density (BMD) [4,5]. Panoramic radiographs were previously used [6] for developing a mandibular cortical index to assess the porosity of cortical bone. Many studies have shown a correlation between the mandibular cortical width (MCW) on dental panoramic radiographs and BMD at the hip [7], lumbar spine [8] and forearm [9], the most common sites of fracture related to osteoporosis in postmenopausal women. The costs associated with advanced imaging techniques and distribution of the equipment limit their accessibility for large segments of populations and broad-based screening examinations.

Recently, computer software-assisted diagnostic techniques have been used because they reduce the influence of subjective human interpretation and have often increased diagnostic accuracy [10]. Osteoporosis screening using a computer-aided diagnosis (CAD) system developed in previous studies needed manual assistance in measuring the MCW [11,12]. Furthermore, MCW measured at only one point (below the mental foramen) may influence measurement error. However, the image of the hyoid bone sometimes overlaps the cortex below the mental foramen on dental panoramic radiographs, which can result in measurement errors with CAD systems such as the one used in the previous study [11]. Continuous measurement of the MCW between the upper and lower boundary at each point below the mental foramen could help to overcome this problem in order to accurately detect osteoporosis [13].

Most research effort on the analysis of orthopaedic X-ray images has been focused on the detection of osteoporosis using methods of texture and fractal analysis [14-16]. Continuous measurement by CAD systems utilizes a trimmed mean method to achieve good diagnostic results. However, using a trimmed mean requires prior threshold setting [13]. Unfortunately, the selection of an initial parameter setting for the prior threshold may affect results drastically. Support vector machine (SVM) methods have the feasibility and superior ability to extract higher-order statistics and have become extremely popular for classification and prediction. As a continuation of our extensive previous work of developing a CAD system [13], we decided to apply the CAD system with SVM-kernel methods to diagnose and classify women with low BMD. This study employs a newly adopted kernel-based radial basis function (RBF)-SVM method instead of a trimmed mean method to improve its diagnostic efficacy and achieve a higher accurate classification rate for the discriminating and identifying women with low BMD from those with normal skeletal BMD. The application of the CAD system used in this study was directed towards problems in classification. The goal of classification was to accurately determine to which set (or class) an unknown item belongs.

## Methods

### Subjects

The Hiroshima University Human Subject Committee approved the study protocol, and dental panoramic radiographs were taken for all the subjects after informed consent was obtained. A total of 531 women underwent skeletal BMD examinations at an oral radiology clinic at Hiroshima University Hospital between 1996 and 2001. This study included 100 postmenopausal women as subjects, of whom 60 were allocated to the training of the tool and 40 to its testing. All 100 women underwent BMD of the lumbar spine (L2-L4) and femoral neck by dual-energy X-ray absorptiometry (DPX-alpha; Lunar Co., Madison, WI, USA). The inclusion criteria were postmenopausal women aged 50 years or older with no previous diagnosis of osteoporosis. The exclusion criteria were subjects who menstruated less than a year, had any metabolic bone disease (hyperparathyroidism, hypoparathyroidism, Paget's disease, osteomalacia, renal osteodystrophy or osteogenesis imperfecta), had cancer with bone metastasis, had significant renal impairment, had history of taking medication known to affect bone metabolism (e.g. oestrogen), had undergone hysterectomy or oophorectomy, had a history of smoking, had any bone destructive lesion (e.g. malignant tumours or osteomyelitis) in the mandible or had any spinal fracture. Spinal fractures were confirmed semi-quantitatively on lateral radiographs.

The subjects were classified as normal (*T-score *> -1.0), osteopenia (*T-score *between -1 and -2.5) or osteoporotic (*T-score *< -2.5) at each skeletal site according to the World Health Organization (WHO) [17] criteria. The Adult Health Study cohort in Japan [18] reported that the cut-off BMD value of osteoporosis in the lumbar spine that was based on the Japanese definition [19] (less than 70%) was similar to that based on the WHO definition (*T-score *< -2.5 SD); therefore, we used the WHO definition in this study.

### Dental panoramic radiography

All of the panoramic radiographs were obtained by using an AZ-3000 Panoramic Dental X-ray (Asahi Co., Kyoto, Japan) at 12 mA and 15 s; kVp values varied between 70 and 80, and a flat-bed scanner (ES-8000; Epson, Tokyo, Japan) was used to digitize the images at a resolution of 300 dpi. Screens of speed group 200 (HG-M; Fuji Photo Film Co., Tokyo, Japan) and film (UR-2; Fuji Photo Film Co.) were used. One set of duplicate films (MI-Dup; Fuji Photo Film Co.) that were processed with an automatic film processor (Cepros M; Fuji Photo Film Co.) comprised the 100 original panoramic radiographs for the assessment. The appearance of the mandibular inferior cortex was clear bilaterally in the radiographs.

### Computerized scheme for automated detection of osteoporosis

**Figure 1:**
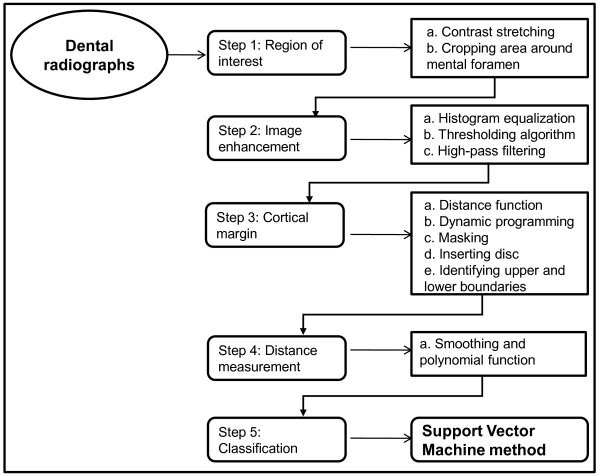
**Schematic diagram depicting the classification of osteoporosis using a support vector machine through continuous cortical width measurement**.

Our osteoporosis detection method is outlined in Figure 1. There are various stages involved in identifying women with low BMD through mandibular cortical width using this CAD system. These include identifying the area of interest, enhancing the original image, locating the inner and outer boundaries of the cortex, measuring the distance between the upper and lower boundaries of the cortical bone and classifying by the SVM method. This system was run on a Pentium [R] Dual-core (CPU 2.50 GHz) with 2 GB of RAM.

### Area of interest determination

**Figure 2:**
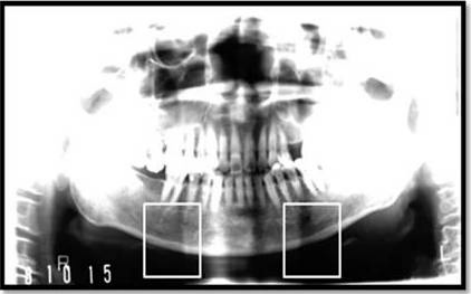
**Digitized dental panoramic radiographs showing two boxes corresponding to the region of interest between the mental foramen and angle of the mandible on the right and left sides of the mandible**.

The mental foramen is one of two holes ('foramina') located on the anterior surface of the mandible. However, the area around the mental foramen is disturbed by low contrast and dark colour. Hence we stretched the appropriate range of intensity values corresponding to the area of interest. Because the original panoramic radiograph had very high resolution, we selected an area of interest for simple and fast computing. The area (300 × 300 pixels) involved in the lower border of the mandibular cortex below the mental foramen, cropped manually on the right and left sides, was considered as the region of interest (Figure 2).

### Image enhancement

The boundary of each object was not sharp, so we removed all areas that were considered to be background and applied enhancement processes to the remaining objects. The typical histogram equalization method is the first step in image enhancement to obtain new enhanced images with a uniform histogram. The most common method of thresholding assigns a pixel to one class if its grey level is greater than a specified threshold and otherwise assigns it to the other class for separating objects from its background.

**Figure 3:**
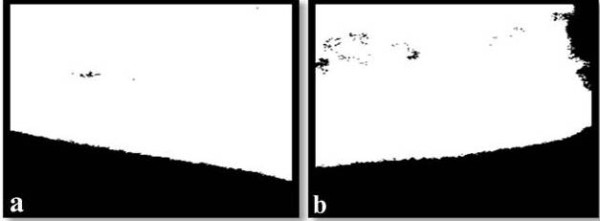
**Binary images of the right (a) and left (b) cortices**.

We applied a thresholding algorithm to classify image pixels into one of two classes; i.e. objects and background, where the threshold is determined on the basis of on intra- and interclass variance of the pixel values [11]. This algorithm generated binary images of the determined area of interest (Figures 3a, b). Multiplying this binary image and the original image removed the background and preserved all grey levels considered to be objects.

We applied an averaging filter as a low-pass filter [20] to the multiplied image and subtracted this low-frequency image from the multiplied image to obtain an image that contained only high frequencies. This step was applied to images that no longer had background illumination variations. Therefore, the high-pass filtering process sharpened the boundary along the cortical bone only because the region of no interest adjacent to the cortical bone had been removed.

**Figure 4:**
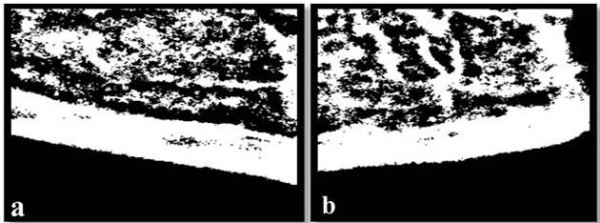
**High-pass filter images of the right (a) and left (b) cortices**.

A threshold related to the mean value of all the pixels in the image was used to binarize the resultant grayscale image (Figures 4a, b).

### Cortical margin determination

**Figure 5:**
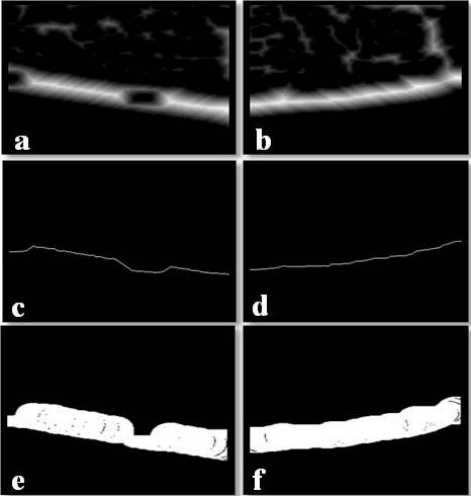
**Images of the right and left sides of the mandibular cortical bone**. Images of eight neighbourhood distance function (a), (b); Images of dynamic programming (c), (d); Images of disc insertion (e), (f).

The cortical margins of the cortex were not clearly delineated because there were some connections to trabecular bones. In some cases, the margins appeared even; however, other margins exhibited defects or porosity. Hence, in this study we employed various image processing techniques [21] for validating the appropriate cortical margin. We applied the eight neighbourhood distance function (ENDF) to analysing the objects with maximum pixels. The ENDF at each pixel represented the distance from the pixel to the margin as the object pixel value (Figures 5a, b). The trace of maximum pixel values represented the medial axis of the cortical bone. This trace was obtained by dynamic programming [22]. Figures 5c and 5d show the trace obtained by applying the dynamic programming method from left to right. The cortical margins were finally obtained as the envelope of the disc, which was located at each pixel on the trace, whose diameter equalled the pixel value (Figures 5e, f).

### Distance measurement

**Figure 6:**
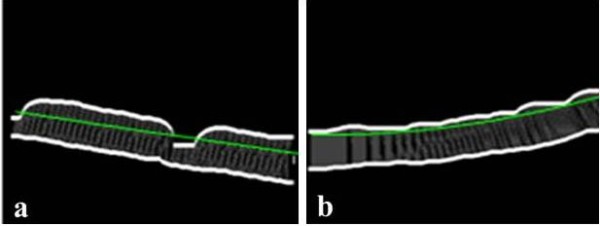
**Smoothing and polynomial images of the right (a) and left (b) cortices**.

This step involved the measurement of the distance between the upper and lower margins of the cortical bone. To determine the direction of the measurements, the least squares method was used to fit a second-order polynomial function to the upper boundary [21]. The cortical width at each point was measured along the line tangential to the polynomial curve, which approximated the upper boundary (Figures 6a, b).

### Classification

We applied a kernel-based SVM to assess the performance of this diagnostic system for achieving high accuracy. While the SVM has been successfully used for medical imaging in several studies, it has not been applied previously for women with low BMD classification on dental panoramic images. It was chosen for its ability to perform well in the presence of noise in the source data and because good separation was achieved in sets of linearly non-separable cases [23,24]. It was proposed that the problem in the original space should be mapped into a higher-dimensional space, presumably making the separation easier in that space (cf. details on the algorithm [25] and details on its implementation, optimization, training and testing [26]). Briefly, both positive and negative examples in a dataset were represented by feature vectors *x_i_*(*i *= 1,2,...,*N*) with corresponding binary labels *y_i_*ε{+1,-1} The SVM algorithm classifies the positive and negative examples by training a classifier that uses a kernel function to map the input samples onto a high-dimensional space that best differentiates the two classes with a maximal margin and a minimal error. The data that lie on the hyperplane margins are support vectors. The decision function for classification of unseen examples is given as

(1) $$f\left(x\right)=\text{sgn}\;\left(\overset{m}{\underset{i=1}{\sum }}\alpha_{i}y_{i}\cdot \text{K}\left(x,x_{i}\right)+b\right)$$

Where K(*x_i_, x_j_*) is the kernel function and the parameters are determined by maximizing the following:

(2) $$\overset{N}{\underset{i=1}{\sum }}\alpha_{i}-\frac{1}{2}\overset{N}{\underset{i=1}{\sum }}\overset{N}{\underset{j=1}{\sum }}\alpha_{i}\alpha_{j}y_{i}y_{j}\text{K}\left(x_{i}\cdot x_{j}\right)$$

under the conditions,

(3) $$\overset{N}{\underset{i=1}{\sum }}\alpha_{i}y_{i}=0and0\leq \alpha_{i}\leq C$$

The variable *C *serves as the cost parameter that controls the trade-off between the margin and classification error. Because the efficacy of the SVM-based classification is dependent on the type of kernel used, we explored the use of various commonly used kernels (linear, sigmoid, polynomial and the RBF) on our datasets. We chose the RBF kernel because it was found to be the most effective.

(4) $$K\left(x_{i},x_{j}\right)=\text{exp}\left(\frac{-||x_{i}-x_{j}|{|}^{2}}{2\gamma^{2}}\right)$$

Two parameters are required to optimize the RBF kernel of the SVM classifier γ that determines the capacity of the RBF kernel and *C*, the regularization parameter. These parameters must be adjusted to obtain the best classification with a reduced number of support vectors because this number is directly related to the speed of execution.

### Statistical analyses

We chose to use the RBF as the kernel function because it was shown to perform well on our datasets for classifications using average and variance results of the continuous measurements between women with low BMD (BMD *T-score *of - 1.0 or less) and normal skeletal BMD. Quadratic programming was used to optimize the combination of parameters and found to yield better classification results at γ = 1 and *C *= 1. Smaller values were used to avoid reproducing noise and avoid over-fitting to the data samples that were used in the training procedure. The RBF parameter and weighting factors were determined by experimentation on the training samples. We employed a data analysis framework written in Matlab, which incorporates freely available SVM tools for Matlab that were implemented by Scholkopf [27], to perform classification. The mean of the MCW on both sides of the mandible was used in this study. The risk-index range that corresponded to a sensitivity of approximately 90% was chosen to determine the optimal cut-off threshold. The sensitivities, specificities, positive predictive values (PPV), negative predictive values (NPV), accuracies and likelihood ratios for the positive (LR+) results for identifying subjects with low BMD were calculated. The accuracy of the classifications of 10 randomly selected subjects for dental panoramic radiographs measured twice with a one-month interval by the same examiner also was evaluated.

## Results and Discussion

The RBF kernel was used to process 60 training sets and classify 40 test sets. For the training sets, the data was classified into an osteoporotic and a normal group and labelled as either '0' or '1' that corresponded to positive and negative examples for SVM training. The RBF kernel-SVM predictions for the diagnostic classification of women had 90.9% sensitivity and 83.8% specificity on the basis of the lumbar spine BMD and 90.0% sensitivity and 69.6% specificity on the basis of the femoral neck BMD (Table 1). The level of performance of the classifications was high except for the low specificity obtained for femoral neck BMD data. The accuracy of the proposed CAD system with SVM for diagnosing women with low BMD at the lumbar spine was 88%, PPV was 71.4% and NPV was 96.7%; on the basis of the femoral neck BMD, the accuracy was 75%, PPV was 46.6% and NPV was 96.0% as presented in Table 2. The overall accuracy of the trimmed mean CAD method [13] for the classifications on the basis of the lumbar spine BMD, was 78%, PPV was 47.4% and NPV was 96.8%, whereas on the basis of the femoral neck BMD, the accuracy was 72%, PPV was 42.9% and NPV was 93.1%. In addition, the sensitivity and specificity for the combined data of both the lumbar spine and femoral neck BMD using SVM method processed with 130 training and 70 testing sets were 90.6% and 80.9% respectively (Table 2). The average time to complete the classification measurement on a single radiograph was only 9.0 s. The accuracy of the classifications for 10 randomly selected subjects that were measured twice with a one-month interval was 90%.

**Table 1:** Performance of the proposed SVM method for identifying women with low lumbar spine BMD and femoral neck BMD at a 95% confidence interval (CI)

Identifying site	Sensitivity %	Specificity %	Positive predictive value %	Negative predictive value %	Accuracy %	Likelihood ratio (+) %
Lumbar spine	90.9 (85.3-96.5)	83.8 (76.6-91.0)	71.4 (62.5-80.3)	96.7 (93.8-99.6)	88.0 (81.6-94.4)	5.5 (4.5-6.5)
Femoral neck	90.0 (84.1-95.9)	69.6 (60.1-78.6)	46.6 (36.8-56.4)	96.0 (92.1-99.2)	75.0 (66.5-83.5)	3.1 (2.2-4.0)

**Table 2:** Performance of the proposed SVM method for identifying women with combined skeletal bone mineral densities (BMD) at a 95% confidence interval (CI)

Identifying site	Sensitivity %	Specificity %	Positive predictive value %	Negative predictive value %	Accuracy %	Likelihood ratio (+) %
Skeletal BMD	90.6 (84.9-96.3)	80.9 (73.2-88.6)	61.2 (51.7-70.8)	96.6 (92.0-100.0)	79.0 (71.0-86.9)	4.1 (3.3-5.1)

We used our proposed SVM method with a CAD system and dental panoramic radiographs to diagnose women with low BMD easily and quickly. The use of the SVM kernel in this study provided a high degree of consistency and reproducibility in the results. One of the key advantages of this CAD system over a manual assessment is the objectivity of the automated evaluation. The proposed CAD system directly assesses the bones on radiographs by measuring the MCW continuously between the mental foramen and mandibular angle, which can reduce measurement errors that occur with the conventional assessment [11]. The trimmed mean method (accuracy = 79%) of the recently proposed CAD method [13] can be replaced by the SVM method (accuracy = 88%) to obtain a better result. The proposed SVM diagnostic model performs differential diagnosis very well. Because classification by the trimmed mean method requires prior threshold setting, the SVM approach for classifying women as having either a low or normal BMD may be a better choice.

In the previously developed CAD systems [11,12], the diagnostic accuracy of the radiologist was adversely affected by manual interactions. Arifin et al. [11] reported that measurement of the MCW at one point (below the mental foramen) by CAD had a detection sensitivity of 88% and specificity of 58.7%. The conventional method of continuous measurements using the trimmed mean technique [13] was reported to have a sensitivity of 90% and specificity of 75% on the basis of the lumbar spine BMD and a sensitivity of 81.8% and specificity of 69.2% on the basis of the femoral neck BMD. However, for our newly proposed SVM method, the sensitivity and specificity were 90.9% and 83.8%, respectively on the basis of the lumbar spine BMD and 90.0% and 69.6%, respectively on the basis of the femoral neck BMD.

These findings indicate that the classification performance of the diagnostic system using the SVM method achieved higher accuracy for detecting women with low BMD compared with the conventional approach. The difference between these results is reasonable because unlike other techniques, the CAD system with SVM has an advantage of converging the problem to the global optimum and not to a local optimum.

Our findings are supported by those of previous studies. Lim et al. [28] evaluated bone fractures from X-ray images with different classifiers and reported a high classification accuracy of 98.2% from a method using a combination of SVM classifiers. Caligiuri et al. [29] showed that their method was promising for discriminating between healthy and fractured bones with high *Az *values. It was also reported that the sensitivity and specificity for an RBF kernel-SVM that was used for the reorganization of nuclear receptors was almost equal to our classification system [30]. Comparisons of our experimental results with those of previous studies demonstrated the feasibility and excellent performance of our proposed system in diagnosing high-risk groups with low BMD or osteoporosis. Several screening tools based on simple questionnaires have been developed to identify postmenopausal women with low skeletal BMD or osteoporosis, and validation of these tools has also been performed in many countries [31]. The sensitivity and specificity of such decision rules in identifying postmenopausal women with osteoporosis ranged from 90% to 92% and 37%-45%, respectively. This proves that the diagnostic efficacy of our SVM method is better than that of the several questionnaire-based screening tools that were used in the previous studies, although the backgrounds of the subjects were different. The limitations in our study were that the number of subjects was relatively small, and the subjects were relatively healthy postmenopausal women because we used rigid exclusion criteria.

## Conclusions

The diagnostic efficacies achieved by the application of an RBF kernel-SVM in our study showed that our CAD system was effective and accurate for identifying women with low BMD. Compared with the previously developed trimmed mean method, the SVM method was shown to be more accurate (88% vs. 79%) and could classify women as having either low or normal BMD more efficiently. On the basis of the highly satisfactory sensitivity and specificity results, the proposed system is expected to be a helpful tool for classifying women with low BMD and can provide a second diagnosis that may reduce misdiagnoses. Our SVM method is considered to be a reliable choice for the proposed system because it is fast and specific for classification, using dental panoramic radiographs, of postmenopausal women with low BMD.

## Competing interests

The authors declare that they have no competing interests.

## Authors' contributions

MSK: Study concept and design; acquisition and interpretation of data and drafting of the manuscript. AA: Study concept and design, analysis and interpretation of data; statistical expertise. AT: Study concept; analysis and interpretation of data; critical revision of the manuscript. TK: Analysis and interpretation of data and statistical assistance. MS: Acquisition, analysis and interpretation of data. All authors read and approved the final manuscript.

## Pre-publication history

The pre-publication history for this paper can be accessed here:

http://www.biomedcentral.com/1471-2342/12/1/prepub
